# Wheat Rhizosphere Metagenome Reveals Newfound Potential Soil Zn-Mobilizing Bacteria Contributing to Cultivars’ Variation in Grain Zn Concentration

**DOI:** 10.3389/fmicb.2021.689855

**Published:** 2021-06-23

**Authors:** Sen Wang, Zikang Guo, Li Wang, Yan Zhang, Fan Jiang, Xingshu Wang, Lijuan Yin, Bo Liu, Hangwei Liu, Hengchao Wang, Anqi Wang, Yuwei Ren, Conghui Liu, Wei Fan, Zhaohui Wang

**Affiliations:** ^1^Guangdong Laboratory for Lingnan Modern Agriculture, Genome Analysis Laboratory of the Ministry of Agriculture and Rural Affairs, Agricultural Genomics Institute at Shenzhen, Chinese Academy of Agricultural Sciences, Shenzhen, China; ^2^State Key Laboratory of Crop Stress Biology in Arid Areas, Northwest A&F University, Yangling, China; ^3^Key Laboratory of Plant Nutrition and Agri-Environment in Northwest China, Ministry of Agriculture, College of Natural Resources and Environment, Northwest A&F University, Yangling, China

**Keywords:** wheat cultivar, rhizosphere, microbiome, soil Zn mobilization, bacteria, Zn biofortification

## Abstract

An effective solution to global human zinc (Zn) deficiency is Zn biofortification of staple food crops, which has been hindered by the low available Zn in calcareous soils worldwide. Many culturable soil microbes have been reported to increase Zn availability in the laboratory, while the status of these microbes in fields and whether there are unculturable Zn-mobilizing microbes remain unexplored. Here, we use the culture-independent metagenomic sequencing to investigate the rhizosphere microbiome of three high-Zn (HZn) and three low-Zn (LZn) wheat cultivars in a field experiment with calcareous soils. The average grain Zn concentration of HZn was higher than the Zn biofortification target 40 mg kg^–1^, while that of LZn was lower than 40 mg kg^–1^. Metagenomic sequencing and analysis showed large microbiome difference between wheat rhizosphere and bulk soil but small difference between HZn and LZn. Most of the rhizosphere-enriched microbes in HZn and LZn were in common, including many of the previously reported soil Zn-mobilizing microbes. Notably, 30 of the 32 rhizosphere-enriched species exhibiting different abundances between HZn and LZn possess the functional genes involved in soil Zn mobilization, especially the synthesis and exudation of organic acids and siderophores. Most of the abundant potential Zn-mobilizing species were positively correlated with grain Zn concentration and formed a module with strong interspecies relations in the co-occurrence network of abundant rhizosphere-enriched microbes. The potential Zn-mobilizing species, especially *Massilia* and *Pseudomonas*, may contribute to the cultivars’ variation in grain Zn concentration, and they deserve further investigation in future studies on Zn biofortification.

## Introduction

Around 20% of the world population are suffering from zinc (Zn) deficiency, and the situation will become worse with the increase of atmosphere carbon dioxide (Myers et al., 2014; Smith and Myers, 2018). An effective solution to address human Zn deficiency is to increase the grain Zn concentration of staple food crops like wheat, namely, Zn biofortification. The target of wheat Zn biofortification is to increase the current grain Zn concentration of 20 ∼ 30 to above 40 mg kg^–1^ that is sufficient for human Zn nutrition (Liu et al., 2014; Chen et al., 2017). Achieving the target of wheat grain Zn biofortification has been hindered by the low soil Zn availability because over 50% of the global wheat growing soils are poor in available Zn (Cakmak and Kutman, 2018). Therefore, it is of great significance to increase the Zn availability in agricultural soils.

Due to the poor mobility of Zn in soil, the absorption of Zn by plant roots mainly occurs in the rhizosphere, where the activities of roots and microorganisms can somewhat increase the amount of available Zn (Rehman et al., 2018). In the calcareous soils distributed worldwide, Zn availability is restricted by alkaline environment and high carbonate content; and various root exudates like carboxylic acids, amino acids, and low-molecular-weight polypeptides can acidify the rhizosphere and solubilize the Zn immobilized in minerals (Rengel, 2015). Besides, the microbes living on root exudates can also produce organic acids, siderophores, and exopolysaccharides that can mobilize micronutrients in rhizosphere soil (Sathya et al., 2016). Inoculation of plant growth-promoting rhizobacteria (PGPR) like *Bacillus*, *Arthrobacter*, and arbuscular mycorrhizal fungi (AMF) has been reported to increase soil available Zn and crop grain Zn concentration, especially in soils with low available Zn (Singh et al., 2018; Coccina et al., 2019). Despite many reports on the isolation and functional verification of soil Zn-mobilizing bacteria, the roles of soil microbes in improving soil Zn availability and promoting crop Zn biofortification remain to be extensively explored.

Previous studies on soil Zn-mobilizing microbes are mainly conducted in the laboratory and focus on the culturable soil Zn-mobilizing microbes, while the natural status of these microbes in fields and whether there are more unculturable Zn-mobilizing microbes are unknown. Recently, the culture-independent metagenomics approach has been used to study the whole rhizosphere microbiome of many plants like wheat (Ofek-Lalzar et al., 2014), barley (Bulgarelli et al., 2015), rice (Zhang et al., 2019), and potato (Shi et al., 2019). It has been widely recognized that rhizosphere microbiome contributes substantially to nutrient uptake (Oldroyd and Leyser, 2020), drought resistance (de Vries et al., 2020), and plant health (Trivedi et al., 2020). Here, we use metagenomic sequencing to investigate the rhizosphere microbiome of six wheat cultivars that exhibit different grain Zn concentrations in a field experiment to (1) characterize the rhizosphere microbiome of high-Zn (HZn) and low-Zn (LZn) wheat; (2) identify the potential soil Zn-mobilizing microbes; and (3) determine the natural abundance of Zn-mobilizing microbes and their contributions to the variation of grain Zn concentration among cultivars. The results show large difference of microbial communities and functions between wheat rhizosphere and bulk soil but small difference between HZn and LZn wheat cultivars. Of the rhizosphere-enriched microbes exhibiting different abundances among cultivars, we identify 30 potential soil Zn-mobilizing bacteria that may contribute to the variation of grain Zn concentration among wheat cultivars.

## Materials and Methods

### Field Experiment

The field experiment was located at the Agriculture Research Station of Northwest A&F University in Yangling, Shaanxi, China (34°18′N, 108°05′E). This site is in the temperate continental monsoon climate zone and located on the southern edge of the Loess Plateau, with elevation of 520 m, annual atmosphere temperature of 12.9°C, annual precipitation of 579 mm, and annual potential evaporation of 1,400 mm. The soil type of the experimental filed is Eum-Orthic Anthrosol according to the soil taxonomy system of the United States Department of Agriculture (USDA), and the soil texture is silt clay loam. Basic soil chemical properties are as follows: pH (water:soil = 2.5:1) 8.23, cation exchange capacity (CEC; 1 M of NaAc) 16.3 cmol kg^–1^, organic matter (0.8 M of K_2_Cr_2_O_7_) 16.3 g kg^–1^, total nitrogen (N) (Kjeldahl) 1.01 g kg^–1^, nitrate-N (1 M of KCl) 15.3 mg kg^–1^, ammonium-N (1 M of KCl) 0.1 mg kg^–1^, total phosphorus (P) (HClO_4_ + H_2_SO_4_) 0.78 g kg^–1^, available P (0.5 M of NaHCO_3_) 11.7 mg kg^–1^, total potassium (K) (NaOH) 14.2 g kg^–1^, available K (1 M of NH_4_Ac) 122 mg kg^–1^, total Zn (HF + HNO_3_ + HClO_4_) 74 mg kg^–1^, and available Zn (0.005 M of DTPA) 0.46 mg kg^–1^.

**FIGURE 1:**
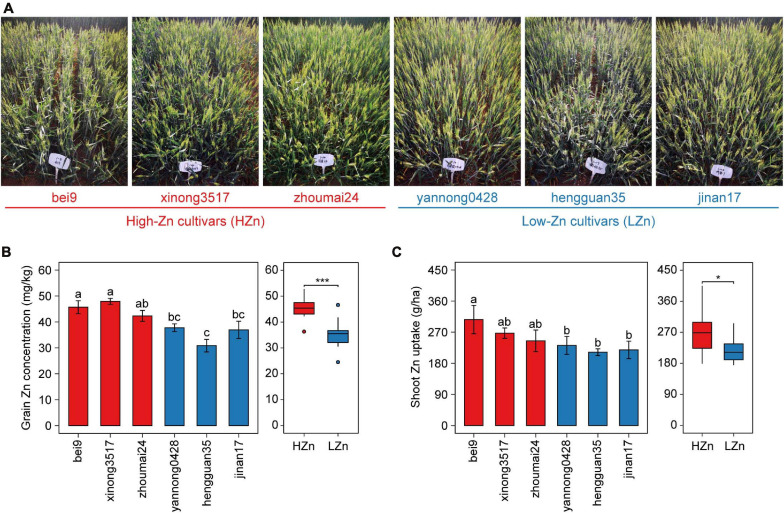
Phenotypic data of the six wheat cultivars grown in calcareous soil. Photographs taken at anthesis **(A)**, grain Zn concentration **(B)**, and shoot Zn uptake **(C)** at maturity of three high-Zn (HZn) and three low-Zn (LZn) cultivars with average grain Zn concentrations higher or lower than the Zn biofortification target 40 mg kg^– 1^. In the left panel of **(B,C)**, the error bars stand for mean ± standard error (*n* = 4), and different lowercase letters indicate significant differences among cultivars (ANOVA, Duncan, *P* < 0.05). In the right panel of **(B,C)**, the inner horizontal lines, and bottom and top of boxes indicate the medians, and 25th and 75th percentiles, respectively; the lower and upper whiskers extend to the minimum and maximum of the data excluding the outliers denoted by dots, respectively; and asterisks indicate significant differences between HZn (*n* = 12) and LZn (*n* = 12) (*t*-test, *P < 0.05, ***P < 0.001).

Based on our previous studies on screening hundreds of wheat cultivars by yield and grain Zn concentration (Wang et al., 2018), six cultivars with similar yields and different grain Zn concentrations were selected for investigation in the present study (Figure 1A). A randomized complete block design was used for the field experiment. Each cultivar had four replicates and was grown with the uniform nutrient application of 150 kg N ha^–1^ as urea (46% N), 100 kg P_2_O_5_ ha^–1^ as calcium superphosphate (16% P_2_O_5_), and 74 kg K_2_O ha^–1^ as potassium sulfate (51% K_2_O). Each plot consisted of five 2-m-long rows with the seed spacing of 2.5 cm and the row spacing of 20 cm. All cultivars were sown on October 3, 2018, and harvested on June 18, 2019. All the fertilizers were applied before sowing, no irrigation was applied during wheat growth period, and only pesticides were used when necessary.

### Plant and Soil Sampling

The top 0- to 20-cm soil of the experimental field was sampled before sowing for the determination of basic soil physicochemical properties. At anthesis, the plant, rhizosphere, and bulk soil samples used for chemical analyses and metagenomic sequencing were collected. For each cultivar, 10 wheat plants were randomly selected and dug out from each plot, cut-off at the stem-root jointing part, and separated into ears, leaves, stems, and roots with adhered rhizosphere soils by sterile stainless tools and shaking. For bulk soil sampling, 10 points were randomly selected from the unplanted areas over 50 cm away from wheat plots in each block and sampled at 0- to 10-cm soil layer and then mixed into one sample using sterile stainless tools. At maturity, the plant samples used for chemical analyses and estimation of yield and biomass were collected. For each cultivar, the wheat plants of 30 ears without roots and soil were sampled by the same procedures used in plant sampling at anthesis, and all the remaining plants were harvested to estimate grain yield.

One third of the samples of root with rhizosphere soil and bulk soil were transported to the laboratory in a dry ice box and stored in an ultra-low-temperature refrigerator at −80°C before metagenomic sequencing, and the other soil and plant samples were transported to the laboratory under air temperature for further processing. Firstly, the rhizosphere soils were separated from roots using a nylon brush; then, the separated rhizosphere soil and bulk soil samples were air-dried and ground to pass through a 1-mm sieve for chemical analysis; finally, the plant samples of separated root, stem, leaf, ear, glume, and grain were successively washed by tap water and distilled water, oven-dried at 65°C and weighed, and ground to powder using a ball miller (Retsch MM400, Haan, Germany) for chemical analysis.

### Physicochemical Analyses

The chemical analyses of soil and plant samples were conducted according to the methods described by Wang et al. (2018). In short, soil pH was measured using CO_2_–free water extraction (water:soil = 2.5:1) and a pH meter, and soil available Zn was extracted by 0.005 M of DTPA-TEA-CaCl_2_ solution and determined by an atomic absorption spectrometer (AAS). Grain yield and biomasses were expressed in oven-dried weight, and plant Zn concentration was determined by microwave digestion with HNO_3_ and H_2_O_2_ and an inductively coupled plasma mass spectrometry (ICP-MS), and then shoot Zn uptake was calculated.

### Metagenomic Sequencing

Rhizosphere soil samples used for metagenomic sequencing were separated from roots according to the methods described by Lundberg et al. (2012) and Donn et al. (2015). Briefly, 2∼3 g of roots with adhered rhizosphere soil were cut into 1- to 2-cm-long pieces and then washed in 30 ml of phosphate-buffered solution (PBS) in a 50-ml sterile tube by continuous shaking at 180 rpm and 4°C for 20 min. Then, the turbid suspension was poured into a new 50-ml tube and centrifuged at 10,000 *g* and 4°C for 20 min to get the rhizosphere soil pellet, which was firstly frozen in liquid nitrogen and then stored at −80°C for DNA extraction. All the above operations were done using sterile tools under sterile conditions. Soil DNA was extracted using the DNeasy PowerSoil DNA Isolation Kit (QIAGEN, Hilden, Germany) according to the provided protocol with minor modifications. In short, 0.4 g of soil was weighed, added into the PowerBead Tube and mixed on a vortex, and incubated at 65°C in a water bath for 10 min before being subjected to the same procedures as described in the protocol. The extracted DNA was quantified by Invitrogen Qubit 4.0 and Qubit dsDNA HS Assay Kit (Thermo Fisher Scientific, Waltham, MA, United States) and quality checked by NanoDrop 2000 (Thermo Fisher Scientific, United States) and agarose gel electrophoresis (Beijing LiuYi Biotechnology, Beijing, China). The DNA concentration was diluted or concentrated to ∼ 25 ng μl^–1^ for the preparation of sequencing library.

The prepared DNA sample was fragmented into 350-bp fragments in 60-μl volume in a microTUBE by Covaris S220 ultrasonicator (Covaris, Woburn, MA, United States). Then, the fragmented DNA was transformed into sequencing library using the Truseq DNA PCR-Free Library Prep Kit (Illumina, San Diego, CA, United States) according to the provided protocol. Finally, the prepared library was sequenced by the Illumina Hiseq X ten (Illumina, United States) in the mode of paired-end 150 bp.

### Bioinformatic Analyses

The raw reads were firstly checked for sequencing quality; and the ambiguous bases, low-quality bases, and adapter contamination were removed using fastp v0.20.0 (Chen et al., 2018). Then the reads were mapped to wheat genome sequence IWGSC RefSeq v1.0 (IWGSC, 2018) using Bowtie 2 v2.2.7 (Langmead and Salzberg, 2012) to remove host contamination and get the clean reads derived from microbes. Afterward, the clean reads were assembled into contigs by MEGAHIT v1.1.3 (Li et al., 2016) with the parameter “–presets meta-large” through three steps: (1) the clean reads of each sample were assembled separately; (2) for each sample, the clean reads were mapped to the assembled contigs by Bowtie 2, and the unmapped clean reads of all samples were pooled and assembled into contigs; and (3) all the contigs of separate and pooled assemblies with length >400 bp were combined to get the final assembly. The genes in assembled contigs were predicted using Prodigal v2.6.3 (Hyatt et al., 2012) with the parameter “-p meta,” and the genes with length >200 bp were clustered by MMseqs2 Linclust (Steinegger and Söding, 2017, 2018) to remove redundant genes with sequence identity >95% and coverage >90%. Then, the non-redundant genes were taxonomically annotated using kaiju v1.7.3 (Menzel et al., 2016) with the parameter “-a greedy” and National Center for Biotechnology Information (NCBI) NR and Taxonomy as reference databases and functionally annotated using eggNOG-Mapper v2.0.1 (Huerta-Cepas et al., 2017) by diamond v0.9.24 (Buchfink et al., 2015) searching against eggNOG 5.0 (Huerta-Cepas et al., 2018) database. The clean reads of each sample were mapped back to the annotated non-redundant gene set by Bowtie 2 to calculate the relative abundances of genes, different taxonomic ranks like phylum and species, and different functional orthologous groups like eggNOG Orthologous Groups (OGs) and categories. The scripts used for bioinformatic analyses are available on https://github.com/kingforest93/Ta6_rhizo_meta_scripts.

To identify the potential soil Zn-mobilizing species, we summarized the functional characteristics of the reported soil Zn-mobilizing microbes in published literature (Supplementary Table 3), selected the OGs relevant to soil Zn mobilization by the functional description, and classified them into three groups as the transmembrane transport of Zn (transporter, export, efflux, etc.), the metabolism of Zn ligands (siderophore, citric acid, etc.), and the promotion of root Zn uptake (growth promotion, auxin, N fixation, etc.) (Supplementary Table 4). Then, the genome sequences of potential soil Zn-mobilizing species were downloaded from NCBI Genome database and subjected to the same functional annotation procedures as described in the annotation of non-redundant genes. The microbes possessing the above functional genes relevant to soil Zn mobilization were regarded as potential soil Zn-mobilizing microorganisms.

### Statistical Analyses

The comparisons of soil pH, soil DTPA-Zn, plant Zn concentration, and Zn uptake among cultivars and bulk soil were conducted by ANOVA and Duncan’s multiple comparison in R 4.0.2 (R Core Team, 2020). The differences of grain Zn concentration and shoot Zn uptake between HZn and LZn group were tested by *t*-test. The within-sample microbial alpha diversity (the Shannon–Wiener index) of species and OGs were analyzed using vegan v2.5-6 package (Oksanen et al., 2019), and its difference between HZn and LZn was tested by *t*-test. The between-sample microbial beta diversity was analyzed by the Bray–Curtis dissimilarity of species and eggNOG OGs, and different samples were ordinated by principal coordinate analysis (PCoA) and non-metric multidimensional scaling (NMDS) using vegan and ggplot2 v3.3.2 package (Wickham, 2016). The relative abundance differences of microbial phyla, species, and OGs between rhizosphere (Rhizo) and bulk soil (Soil) and between HZn and LZn were tested by Wilcoxon’s rank-sum test, and *P*-values were adjusted by the Benjamini–Hochberg (BH) method (Benjamini and Hochberg, 1995). Spearman’s correlations between plant Zn concentration and Zn uptake and the relative abundances of soil Zn-mobilizing microbial species were analyzed using psych package (Revelle, 2013). The co-occurrence network of rhizosphere-enriched microbes soil Zn-mobilizing microbes based on their Spearman’s correlations were constructed using igraph package (Csardi and Nepusz, 2005). All figures were drawn in R 4.0.2 and Adobe Illustrator CC 2017. The scripts used for statistical analyses and graphing are available on https://github.com/kingforest93/Ta6_rhizo_meta_scripts.

## Results

### Rhizosphere Metagenome Assembly and Annotation of Wheat Cultivars With Various Grain Zn Concentrations

The six wheat cultivars exhibited significant differences in grain Zn concentration and shoot Zn uptake when grown on calcareous soils (ANOVA, *P* < 0.05, Figures 1B,C), consistent with the previous studies on these cultivars (Wang et al., 2018). Based on the average grain Zn concentration higher or lower than the wheat Zn biofortification target 40 mg kg^–1^, the six cultivars were classified into HZn and LZn groups, and the grain Zn concentration and shoot Zn uptake of HZn wheat were significantly higher than those of LZn wheat (*t*-test, *P* < 0.05, Figures 1B,C). Metagenomic sequencing of the 24 rhizosphere soil samples of six cultivars and four bulk soil samples generated a total of 583.4-Gb raw reads and 444.3-Gb clean reads without low-quality bases and adapter and host contamination (Table 1 and Supplementary Table 1). The clean reads were assembled into 166,876,821 contigs with N50 of 631 bp, and the average read mapping rate to assembly was 87.75% (Table 1 and Supplementary Table 1), higher than the previously reported rhizosphere metagenome of wheat (49.2%) and cucumber (70.8%) (Ofek-Lalzar et al., 2014) and citrus (48.3%) (Xu et al., 2018).

**TABLE 1:** Summary of wheat rhizosphere metagenome sequencing, assembly, and annotation.

Item	Value
Number of samples	28
Total raw reads	1,944,609,559
Total raw bases (bp)	583,382,867,700
Total host-free clean reads	1,597,183,845
Total host-free clean bases (bp)	444,251,807,465
Total assembled contigs (>400 bp)	166,876,821
Total contig length (bp)	109,923,227,901
Contig N50 (bp)	631
Reads mapped to contigs (%)	87.75 ± 0.42
Non-redundant genes (>200 bp)	86,919,970
Average gene length (bp)	490
Reads mapped to genes (%)	81.11 ± 0.56
Genes with taxonomy annotation (%)	83.89
Genes with eggNOG OGs (%)	84.07

To get the taxonomic and functional information of wheat rhizosphere microbiome, the genes in the assembled contigs were predicted and clustered to generate a total of 86,919,970 non-redundant genes with the average length of 490 bp and the average read mapping rate of 81.11% (Table 1 and Supplementary Table 1). Of the non-redundant genes, 83.89% had taxonomy annotation, and 79.21, 48.59, and 20.71% were taxonomically classified to 84 phyla, 1,023 families, and 13,254 species (Supplementary Table 2), respectively. Besides, 84.07% of the non-redundant genes had functional annotation, and 84.07 and 53.37% were functionally annotated with 1,572,349 eggNOG OGs and 15,180 KEGG KOs (Table 1 and Supplementary Table 2), respectively. Therefore, the constructed gene set here provides the foundation for analyzing the rhizosphere microbial communities and functions of winter wheat grown in calcareous soils.

### Wheat Rhizosphere and Bulk Soil Exhibit Large Difference in Microbial Taxonomic and Functional Profiles

**FIGURE 2:**
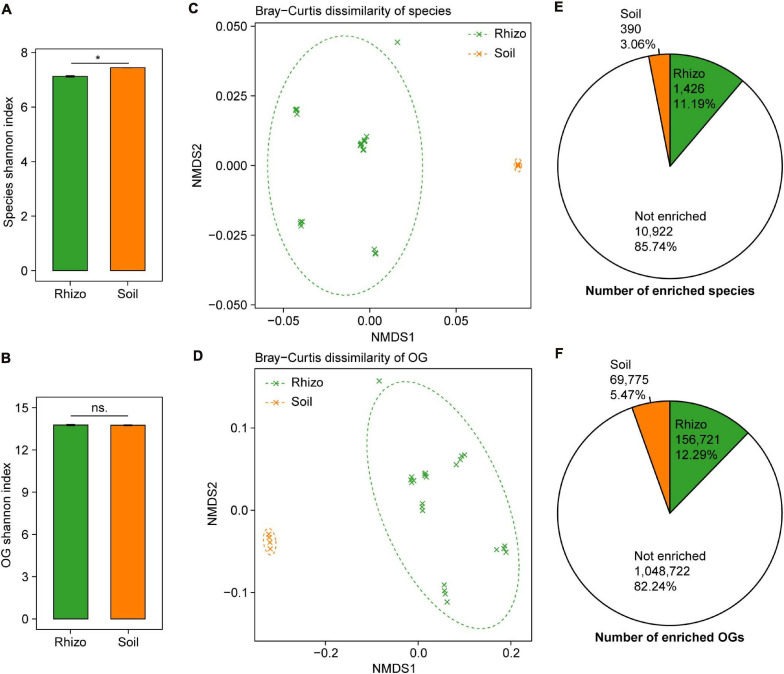
Comparisons of microbial taxonomic and functional diversity between wheat rhizosphere (Rhizo) and bulk soil (Soil). Within-sample alpha diversity (the Shannon–Wiener index) of microbial species **(A)** and eggNOG Orthologous Groups (OGs) **(B)** between Rhizo and Soil. The error bars stand for mean ± standard error (Rhizo, *n* = 24; Soil, *n* = 4), and asterisks indicate significant differences between Rhizo and Soil (*t*-test, *P < 0.05; ns, not significant). Between-sample beta diversity of species **(C)** and OGs **(D)** among Rhizo and Soil samples are analyzed by non-metric multidimensional scaling (NMDS) of the Bray–Curtis dissimilarity. Green and orange crosses refer to Rhizo and Soil samples, respectively; and the corresponding ellipses cover 90% of the data range. The number of species **(E)** and OGs **(F)** enriched in Rhizo (green pie, relative abundance ratio of Rhizo/Soil >1.5), Soil (orange pie, Rhizo/Soil <0.5) [Wilcoxon rank sum test, the Benjamini–Hochberg (BH) adjusted *P* < 0.05], and neither enriched.

The plant rhizosphere microbiome has been regarded as a subset of the bulk soil microbiome, and we compared the microbial taxonomic and functional diversity between wheat rhizosphere and bulk soil. The microbial taxonomic alpha diversity (species Shannon index) of wheat rhizosphere was 4.3% lower than that of bulk soil (*t*-test, *P* < 0.05, Figure 2A). Of the major phyla in both wheat rhizosphere and bulk soil, Proteobacteria and Bacteroidetes showed significantly higher relative abundances in wheat rhizosphere, while Acidobacteria showed significantly higher abundances in bulk soil (Wilcoxon rank-sum test, BH adjusted *P* < 0.05, Supplementary Figure 2E), consistent with the recent studies on wheat rhizosphere microbiome (Illescas et al., 2020; Rossmann et al., 2020). The microbial functional alpha diversity (eggNOG OG Shannon index) showed no significant difference between rhizosphere and bulk soil (Figure 2B), and the relative abundances of most eggNOG functional categories were also similar between rhizosphere and bulk soil (Supplementary Figure 2F). Due to the high functional redundancy in soil microbial communities (Banerjee et al., 2016), possibly less diverse microbial communities in rhizosphere can carry out the similar diverse functions to that in bulk soil.

There were high microbial taxonomic and functional beta diversity between wheat rhizosphere and bulk soil, as shown by the clearly separated rhizosphere and bulk soil samples in both PCoA and NMDS ordination plots based on the Bray–Curtis dissimilarity of species and eggNOG OG (Figures 2C,D and Supplementary Figures 2A,B). Comparisons of the relative abundances of species and OG between rhizosphere and bulk soil showed that 1,426 species and 156,721 OGs were significantly enriched in rhizosphere with the abundance ratio of rhizosphere to bulk soil >1.5 (Wilcoxon rank-sum test, BH adjusted *P* < 0.05, Figures 2E,F and Supplementary Figures 2C,D). In contrast, 390 species and 69,775 OGs were significantly enriched in bulk soil with the abundance ratio of rhizosphere to bulk soil <0.5 (Wilcoxon rank-sum test, BH adjusted *P* < 0.05, Figures 2E,F and Supplementary Figures 2C,D). Many of the rhizosphere-enriched species are widely reported plant growth-promoting microorganisms, such as the species of *Bacillus*, *Burkholderia*, *Glomus*, *Pseudomonas*, *Rhizobium*, *Rhizophagus*, and *Trichoderma* (de Souza et al., 2015; Rosier et al., 2018). Besides, 17 of 38 previously reported soil Zn-mobilizing microbial genera or species were also among the rhizosphere-enriched microbes (Supplementary Table 3). Therefore, wheat roots may recruit a subset of soil microbes in the rhizosphere to promote plant growth and nutrient uptake.

### Small Difference of Rhizosphere Microbiome Exists Between High-Zn and Low-Zn Wheat

**FIGURE 3:**
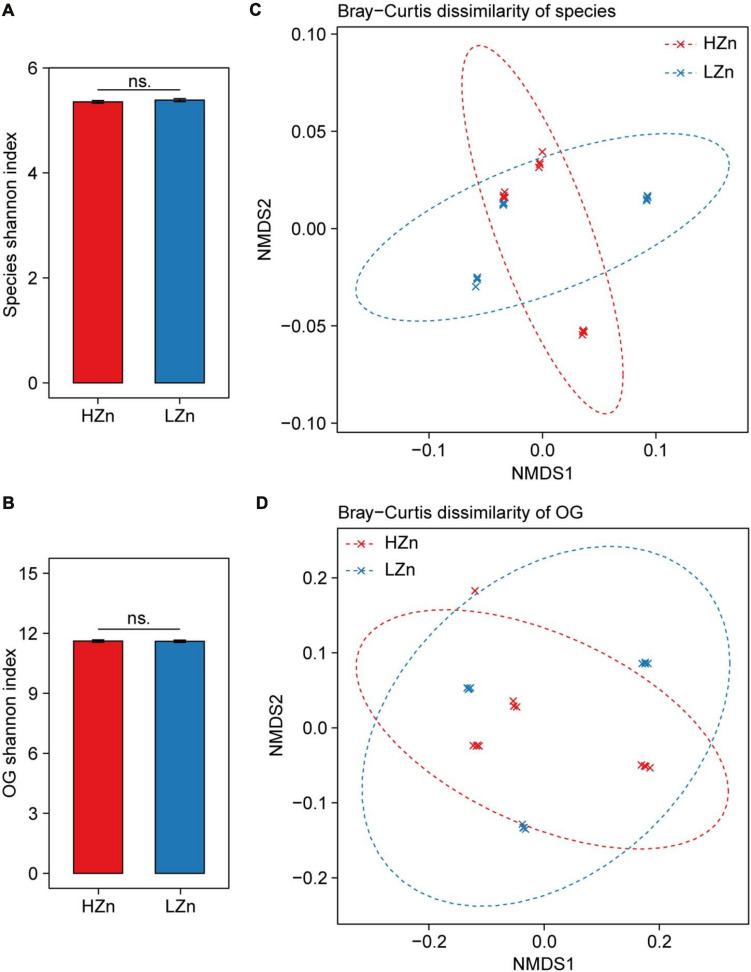
Comparisons of rhizosphere microbiome diversity between high-Zn (HZn) and low-Zn (LZn) wheat. Within-sample alpha diversity (the Shannon–Wiener index) of rhizosphere-enriched species **(A)** and eggNOG Orthologous Groups (OGs) **(B)** between HZn and LZn. The error bars stand for mean ± standard error (HZn, *n* = 12; LZn, *n* = 12); and ns indicates no significant difference between HZn and LZn (*t*-test, *P* < 0.05). Between-sample beta diversity of rhizosphere-enriched species **(C)** and OGs **(D)** among HZn and LZn samples are analyzed by non-metric multidimensional scaling (NMDS) of the Bray–Curtis dissimilarity. Red and blue crosses refer to HZn and LZn samples, respectively; and the corresponding ellipses cover 90% of the data range.

Compared with the large difference of microbial diversity between rhizosphere and bulk soil, small difference of rhizosphere microbiome was observed between HZn and LZn wheat. The microbial alpha diversity (Shannon index) of rhizosphere-enriched species and eggNOG OGs showed no significant difference between HZn and LZn (Figures 3A,B). Possibly, many of the rhizosphere-enriched microbes provides beneficial functions necessary for both HZn and LZn wheat cultivars. Although the microbial beta diversity of rhizosphere-enriched species and OGs between HZn and LZn were also small, there were still some differences among HZn and LZn cultivars as shown by the non-overlapping sample points in both PCoA and NMDS ordination plots based on the Bray–Curtis distance (Figures 3C,D and Supplementary Figures 3A,B). Thus, it is necessary to further analyze the difference of each species and OG between HZn and LZn rhizosphere.

#### High-Zn Wheat Recruit More Bacteria Relevant to Soil Zn Mobilization in the Rhizosphere

**FIGURE 4:**
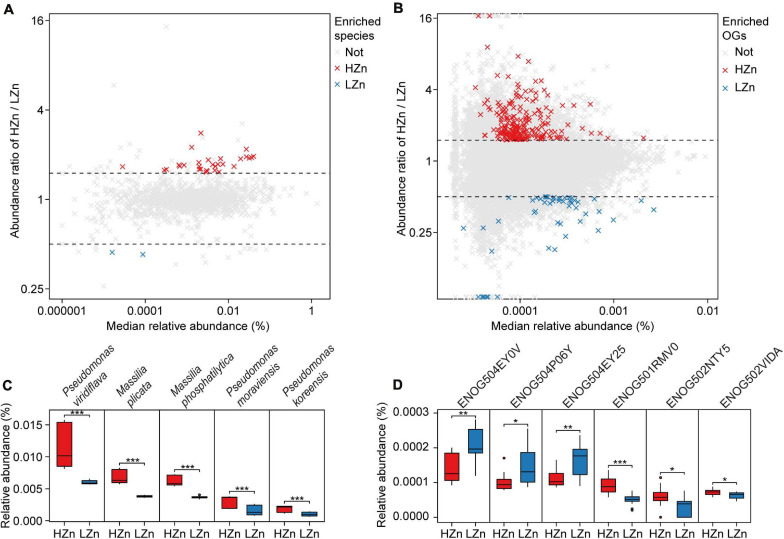
Comparisons of the rhizosphere microbes and functions relevant to soil Zn mobilization between high-Zn (HZn) and low-Zn (LZn) wheat. The median relative abundance and abundance ratio (HZn/LZn) of microbial species **(A)** and eggNOG Orthologous Groups (OGs) **(B)** enriched in the rhizosphere of HZn (ratio >1.5, red crosses) or LZn (ratio <0.5, blue crosses) [Wilcoxon rank sum test, the Benjamini–Hochberg (BH) adjusted *P* < 0.05] and neither enriched (light gray crosses). Comparisons of the relative abundances of representative HZn or LZn-enriched species **(C)** and OGs **(D)** relevant to soil Zn mobilization between HZn and LZn. The inner horizontal lines, and bottom and top of boxes indicate the medians, and 25th and 75th percentiles, respectively; the lower and upper whiskers extend to the minimum and maximum of the data excluding the outliers denoted by dots, respectively; and asterisks indicate significant differences between HZn (*n* = 12) and LZn (*n* = 12) [Wilcoxon rank sum test, the Benjamini–Hochberg (BH) adjusted, *P < 0.05, **P < 0.01, ***P < 0.001].

Comparisons of the relative abundances of rhizosphere-enriched eggNOG OGs between HZn and LZn wheat showed that 368 OGs were significantly enriched in HZn with the abundance ratio of HZn to LZn >1.5 and 86 OGs were enriched in LZn with the abundance ratio of HZn to LZn <0.5 (Wilcoxon rank-sum test, BH adjusted *P* < 0.05, Figure 4B). Of these OGs, 16 HZn-enriched and 16 LZn-enriched OGs are relevant to soil Zn mobilization by comparing their functional descriptions with the functional characteristics of previously reported soil Zn-mobilizing microbes (Supplementary Tables 3, 5). The 16 HZn-enriched OGs are mainly involved in the synthesis of Zn ligands like malate (ENOG502NTY5), siderophore (ENOG502VIDA), and auxin export (ENOG501RMV0) that can promote soil Zn mobilization, while the 16 LZn-enriched OGs are mainly involved in the lysis of Zn ligands like citrate (ENOG504EY0V and ENOG504EY25) that are unfavorable to soil Zn mobilization (Figure 4D and Supplementary Table 5). Hence, HZn wheat cultivars may recruit more functional genes relevant to Zn mobilization to help increase the Zn availability in rhizosphere soil.

Comparisons of the relative abundances of rhizosphere-enriched species between HZn and LZn wheat found that 30 species were significantly enriched in HZn with the abundance ratio of HZn to LZn >1.5 and two species were enriched in LZn with the abundance ratio of HZn to LZn <0.5 (Wilcoxon rank-sum test, BH adjusted *P* < 0.05, Figure 4A and Supplementary Table 6). Of the 32 species, three HZn-enriched species belong to the reported soil Zn-mobilizing genus *Pseudomonas*, which is one of the 38 previously published Zn-mobilizing microbial species or genera (Supplementary Table 3). Nearly half of the previously reported Zn-mobilizing microbes were enriched in wheat rhizosphere, but most of them showed no significant difference between HZn and LZn (Supplementary Figures 4A,B and Supplementary Table 3). Differently, the 32 HZn- or LZn-enriched species were also enriched in wheat rhizosphere, and they exhibited significant differences between HZn and LZn, such as the much higher abundances of *Pseudomonas* and *Massilia* species in HZn than those in LZn (Wilcoxon rank-sum test, BH adjusted *P* < 0.05, Figure 4C and Supplementary Table 6). Besides, genome functional annotation of the 32 species showed that 28 HZn-enriched and two LZn-enriched species possess the functional genes involved in soil Zn mobilization (Table 2). Therefore, the previously reported Zn-mobilizing microbes can promote the Zn uptake of all wheat plants, while the identified 30 HZn- or LZn-enriched species may be the potential soil Zn-mobilizing microbes contributing to the variations of shoot Zn uptake and grain Zn concentration among cultivars.

**TABLE 2:** Functional statistics of the potential soil Zn-mobilizing microbes enriched in wheat rhizosphere.

Species name	Genome size (Mbp)	Total coding genes	Number of the genes involved in^a^			Verified in published literature^b^
			Zn import and export	Zn ligand metabolism	Zn uptake by root	
*Abiotrophia defectiva*	2.05	1,819	4	4	1	No
*Adhaeribacter aerolatus*	6.68	5,177	2	16	1	No
*Adhaeribacter aquaticus*	5.17	4,445	1	15	0	No
*Dietzia kunjamensis*	3.54	3,207	4	12	1	No
*Duganella radicis*	6.99	6,039	2	28	1	No
*Duganella sacchari*	6.68	5,833	2	25	1	No
*Herminiimonas arsenicoxydans*	3.42	3,186	2	24	1	No
*Herminiimonas fonticola*	3.11	2,901	3	24	1	No
*Janthinobacterium agaricidamnosum*	5.95	5,067	2	26	1	No
*Janthinobacterium lividum*	6.37	5,533	1	24	1	No
*Janthinobacterium psychrotolerans*	5.84	5,185	2	23	1	No
*Janthinobacterium svalbardensis*	6.27	5,431	1	23	1	No
*Massilia armeniaca*	6.34	5,240	2	21	1	No
*Massilia buxea*	6.36	5,376	3	24	1	No
*Massilia eurypsychrophila*	5.95	5,316	2	24	1	No
*Massilia glaciei*	6.29	5,367	3	19	1	No
*Massilia namucuonensis*	8.49	7,126	3	42	2	No
*Massilia phosphatilytica*	7.18	6,178	2	33	1	No
*Massilia plicata*	5.89	4,913	2	21	1	No
*Massilia psychrophila*	4.73	4,165	3	21	1	No
*Massilia timonae*	5.97	5,076	3	21	1	No
*Massilia violaceinigra*	7.53	6,291	2	19	1	No
*Pandoraea eparura*	5.21	4,532	5	35	1	No
*Pseudoduganella danionis*	5.14	4,351	2	21	1	No
*Pseudoduganella eburnea*	6.04	5,624	1	23	1	No
*Pseudoduganella violaceinigra*	6.11	5,636	2	24	1	No
*Pseudomonas koreensis*	6.12	5,435	14	25	1	Yes
*Pseudomonas moraviensis*	6.12	5,393	13	26	1	Yes
*Pseudomonas viridiflava*	6.09	5,283	13	25	1	Yes
*Rugamonas rubra*	6.83	5,916	3	26	2	No

*^*a*^The genes are classified by the eggNOG OGs that they belong to into three functional groups as presented in Supplementary Table 4*

*^*b*^The isolated soil Zn-mobilizing microbes in published literature and their abundances are summarized in Supplementary Table 3.*

### Potential Soil Zn-Mobilizing Bacteria Are Highly Related With the Variation of Wheat Grain Zn Concentration

The relationships of the 30 potential soil Zn-mobilizing species and 25 published Zn-mobilizing species with the variation of Zn uptake and concentration among wheat cultivars were analyzed by Spearman’s correlation. Results showed that 23 potential Zn-mobilizing species were positively correlated with wheat grain Zn concentration, while only two of the 30 species were negatively correlated with grain Zn concentration (Table 3). Differently, most of the previously published Zn-mobilizing species showed weak correlations with wheat Zn uptake and grain Zn concentration (Table 3). Thus, the identified potential Zn-mobilizing bacteria, especially the species of genera *Adhaeribacter*, *Janthinobacterium*, *Massilia*, and *Pseudomonas*, may contribute to the variation of wheat grain Zn concentration among different cultivars.

**TABLE 3:** Spearman’s correlations between the relative abundances of soil Zn-mobilizing microbes and wheat grain Zn concentration and shoot Zn uptake at maturity.

Potential Zn-mobilizing species	Shoot Zn uptake	Grain Zn concentration	Published Zn-mobilizing species	Shoot Zn uptake	Grain Zn concentration
*Abiotrophia defectiva*	–0.393	−0.705*	*Azospirillum brasilense*	–0.014	–0.209
*Adhaeribacter aerolatus*	0.202	0.358	*Azospirillum lipoferum*	–0.033	–0.247
*Adhaeribacter aquaticus*	0.262	0.494*	*Azotobacter chroococcum*	0.044	0.069
*Dietzia kunjamensis*	–0.346	−0.485*	*Bacillus anthracis*	–0.285	–0.096
*Duganella radicis*	0.368	0.715*	*Bacillus aryabhattai*	–0.061	–0.104
*Duganella sacchari*	0.313	0.623*	*Bacillus cereus*	0.117	0.181
*Herminiimonas arsenicoxydans*	0.441	0.440	*Bacillus megaterium*	–0.256	–0.381
*Herminiimonas fonticola*	0.324	0.283	*Bacillus safensis*	0.036	–0.107
*Janthinobacterium agaricidamnosum*	0.392	0.610*	*Bacillus subtilis*	0.126	–0.209
*Janthinobacterium lividum*	0.299	0.676*	*Bacillus tequilensis*	–0.105	–0.010
*Janthinobacterium psychrotolerans*	0.270	0.500*	*Bacillus thuringiensis*	–0.003	0.377
*Janthinobacterium svalbardensis*	0.394	0.581*	*Burkholderia cepacia*	0.268	0.442
*Massilia armeniaca*	0.431	0.563*	*Enterobacter cancerogenus*	–0.029	0.247
*Massilia buxea*	0.274	0.570*	*Enterobacter ludwigii*	0.096	0.040
*Massilia eurypsychrophila*	0.337	0.734*	*Klebsiella pneumoniae*	0.015	–0.310
*Massilia glaciei*	0.360	0.697*	*Microbacterium saperdae*	0.024	0.057
*Massilia namucuonensis*	0.246	0.665*	*Pseudarthrobacter chlorophenolicus*	0.360	0.111
*Massilia phosphatilytica*	0.383	0.581*	*Pseudarthrobacter sulfonivorans*	0.243	–0.015
*Massilia plicata*	0.294	0.562*	*Pseudomonas aeruginosa*	0.284	0.231
*Massilia psychrophila*	0.340	0.682*	*Pseudomonas fluorescens*	0.133	0.517
*Massilia timonae*	0.353	0.503*	*Pseudomonas monteilii*	–0.036	0.184
*Massilia violaceinigra*	0.268	0.697*	*Pseudomonas putida*	0.448	0.509
*Pandoraea eparura*	0.599*	0.816*	*Rhizophagus irregularis*	–0.184	–0.325
*Pseudoduganella danionis*	0.254	0.550*	*Stenotrophomonas chelatiphaga*	–0.245	–0.011
*Pseudoduganella eburnea*	0.224	0.554*	*Trichoderma harzianum*	–0.157	–0.369
*Pseudoduganella violaceinigra*	0.293	0.578*			
*Pseudomonas koreensis*	0.180	0.385			
*Pseudomonas moraviensis*	0.212	0.404			
*Pseudomonas viridiflava*	0.416	0.557*			
*Rugamonas rubra*	0.304	0.579*			

*Significant correlations at the Benjamini–Hochberg (BH) adjusted *P* < 0.05.

**FIGURE 5:**
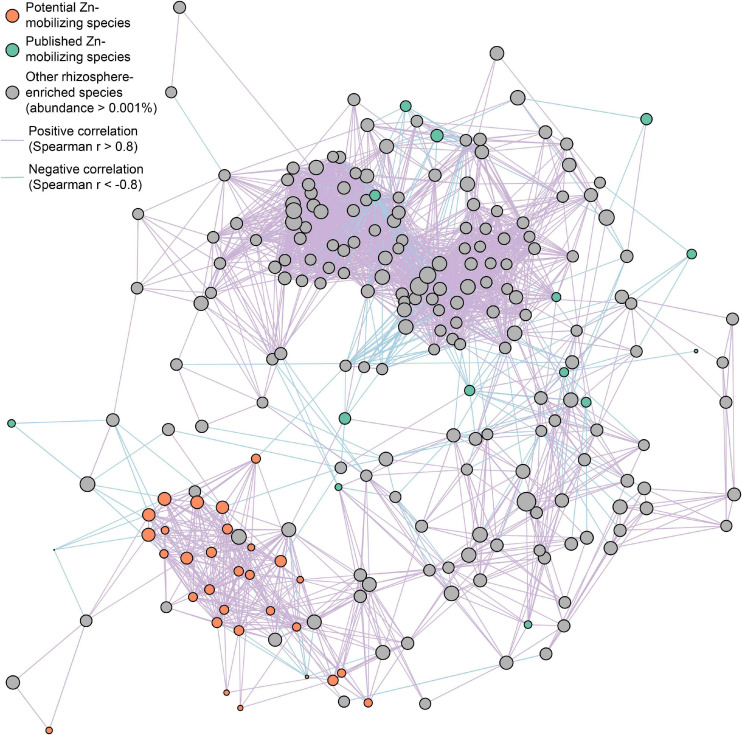
Co-occurrence network of potential (orange circles) and published (green circles) soil Zn-mobilizing microbial species and other rhizosphere-enriched species (gray circles) with median relative abundances above 0.001% in high-Zn and low-Zn wheat cultivars. The size of each circle is proportional to the median relative abundance of the corresponding microbial species in wheat rhizosphere. The purple and blue lines linking circles refer to highly positive (*r* > 0.8) and negative (*r* < –0.8) Spearman’s correlations, respectively, between the relative abundances of the corresponding species.

Furthermore, the interspecies relationships of soil Zn-mobilizing species with other abundant rhizosphere-enriched microbes (abundance >0.001%) in wheat rhizosphere were analyzed by Spearman’s correlation of their relative abundances and illustrated in a co-occurrence network (Figure 5). Considering the strong correlations (| *r*| > 0.8) among species, most of the relatively abundant potential Zn-mobilizing species closely related with each other and formed a module in the network, while the published Zn-mobilizing species scattered in the network (Figure 5). Besides, both the potential and published Zn-mobilizing species had many links with other rhizosphere-enriched microbes in the network (Figure 5). Therefore, the relatively abundant potential Zn-mobilizing species like *Adhaeribacter*, *Janthinobacterium*, *Massilia*, and *Pseudomonas* could cooperate with many rhizosphere-enriched microbes and form a local community to mobilize nutrients and promote the plant growth of wheat.

## Discussion

Host plant genetic factors are thought to have much weaker influences on shaping rhizosphere microbiome than compartment niches do (Xiong et al., 2020). We provide more evidence to this by showing the large difference of microbial communities and functions between wheat rhizosphere and bulk soil but small difference among wheat cultivars (Figures 2, 3), similar to the previous studies on the rhizosphere microbiome of wheat, maize, barley, oat, bean, tomato, and cucumber (Turner et al., 2013; Ofek et al., 2014). Many of the rhizosphere microbes can help host plants fight against plant pathogens (Trivedi et al., 2020), cope with abiotic stresses like drought (de Vries et al., 2020), and absorb nutrients from soil (Oldroyd and Leyser, 2020); and these benefits are necessary for all plants regardless of cultivar or genotype. As observed in this study, many of the rhizosphere-enriched previously reported soil Zn-mobilizing microbes can promote the Zn mobilization and uptake in both HZn and LZn wheat cultivars.

Inoculation of soil microorganisms has been widely reported to improve plant Zn uptake by solubilizing unavailable Zn in soil or promoting the Zn absorption by roots (Rehman et al., 2018). The mechanisms of improving soil Zn availability by microorganisms mainly include three ways: (1) exudate organic acids or protons to acidify soil to solubilize unavailable Zn-containing minerals; (2) release siderophores, nicotinamides, etc., to chelate with Zn ions fixed in soil minerals and metal oxides and make this part of Zn mobile; and (3) produce auxins or fix nitrogen to promote the growth of plant roots and the absorption of Zn from soil (Supplementary Table 3; Sathya et al., 2016; Rehman et al., 2018). In the present study, all the 30 newfound potential soil Zn-mobilizing bacteria possess the genes involved in soil Zn mobilization, in particular the synthesis and exudation of Zn ligands like organic acids and chelates, and may contribute to the cultivars’ variation in grain Zn concentration (Figure 4 and Tables 2, 3). Among the 30 potential soil Zn-mobilizing bacteria, *Pseudomonas* has been reported to produce citric acids (Saleh et al., 2019) and siderophores (Bultreys and Gheysen, 2000) that can solubilize unavailable Zn in soil (Duffner et al., 2012; Oburger et al., 2014), and *Massilia* has been reported to fix N (Feng et al., 2015) and produce organic acids (Zheng et al., 2017) that can promote the growth and Zn uptake by roots. Therefore, the various abundance of newfound potential Zn-mobilizing bacteria, especially *Massilia* and *Pseudomonas* species, may partly contribute to the different grain Zn concentrations among wheat cultivars.

Due to the enormous complex microbial community in soil and plant rhizosphere, a single microbe usually performs a small part of complex functions like nutrient mobilization. In plant rhizosphere, a group of microbes are often necessary to generate effective and stable plant growth promotion effects in agricultural system (Vorholt et al., 2017; Toju et al., 2018). Here, we find that most of the potential soil Zn-mobilizing microbes form a local community with strong positive interspecies relationships, and they may cooperate with other plant growth-promoting species in wheat rhizosphere (Figure 5). In other words, the newfound potential Zn-mobilizing species, in particular *Massilia* and *Pseudomonas* species, may constitute a small functional module to promote soil Zn mobilization and contribute to the various grain Zn concentrations among wheat cultivars. In the future, the 30 newfound potential soil Zn-mobilizing bacteria deserve to be isolated and cultured with the development of high-throughput targeted culturomics technology. Furthermore, the isolates with strong abilities to increase soil Zn availability can be used as a synthetic community to promote the nutrient uptake and growth performance of cereal crops.

## Conclusion

In conclusion, analyses of the rhizosphere metagenome of six wheat cultivars that exhibit different grain Zn concentrations in calcareous soils reveal large difference in the microbial communities and functions between wheat rhizosphere and bulk soil but small difference between HZn and LZn cultivars. The rhizosphere-enriched microbes include many PGPR and previously reported soil Zn-mobilizing microbes, which may promote the growth and nutrient uptake of both HZn and LZn wheat cultivars. Thirty rhizosphere-enriched bacteria are identified as newfound potential soil Zn-mobilizing microbes, which exhibit different relative abundances between HZn and LZn and possess the functional genes involved in soil Zn mobilization or promoting root Zn uptake, especially the synthesis and exudation of organic acids and siderophores. Most of the potential Zn-mobilizing microbes are positively correlated with grain Zn concentration and may constitute a local community to promote soil Zn mobilization. The variation of potential Zn-mobilizing species, especially *Massilia* and *Pseudomonas*, may be the source of variation in grain Zn concentration among wheat cultivars, and they deserve further isolation and verification in future studies on Zn biofortification of cereal crops.

## Data Availability Statement

The generated reads in the present study have been deposited in NCBI SRA under BioProject accession PRJNA688820 (SRR3336283 to SRR3336319) and National Genomics Data Center and BIG Data Center (NGDC BIG) under BioProject accession PRJCA003344 (CRR196125 to CRR196152).

## Author Contributions

SW: conceptualization, data curation, formal analysis, investigation, methodology, visualization, and writing (draft and revision). ZG and LW: data curation, investigation, methodology, resources, visualization, and writing (revision). YZ: formal analysis, methodology, resources, and visualization. FJ: formal analysis, methodology, software, and visualization. XW: data curation, investigation, methodology, and resources. LY: formal analysis, methodology, resources, and visualization. BL, HL, HW, and AW: formal analysis, resources, software, and visualization. YR and CL: methodology, investigation, and resources. WF: conceptualization, funding acquisition, methodology, project administration, supervision, visualization, and writing (revision). ZW: conceptualization, funding acquisition, project administration, resources, supervision, and writing (revision). All authors contributed to the article and approved the submitted version.

## Conflict of Interest

The authors declare that the research was conducted in the absence of any commercial or financial relationships that could be construed as a potential conflict of interest.
