# Supplementary material for: Wheat Rhizosphere Metagenome Reveals Newfound Potential Soil Zn-Mobilizing Bacteria Contributing to Cultivars’ Variation in Grain Zn Concentration
Source: Front Microbiol. 2021 Jun 23;12:689855. doi: 10.3389/fmicb.2021.689855 (PMC8261137; doi:10.3389/fmicb.2021.689855)
Supplement: Supplementary file 7 [file Data_Sheet_1.docx]

Supplementary Material

# Supplementary Figures


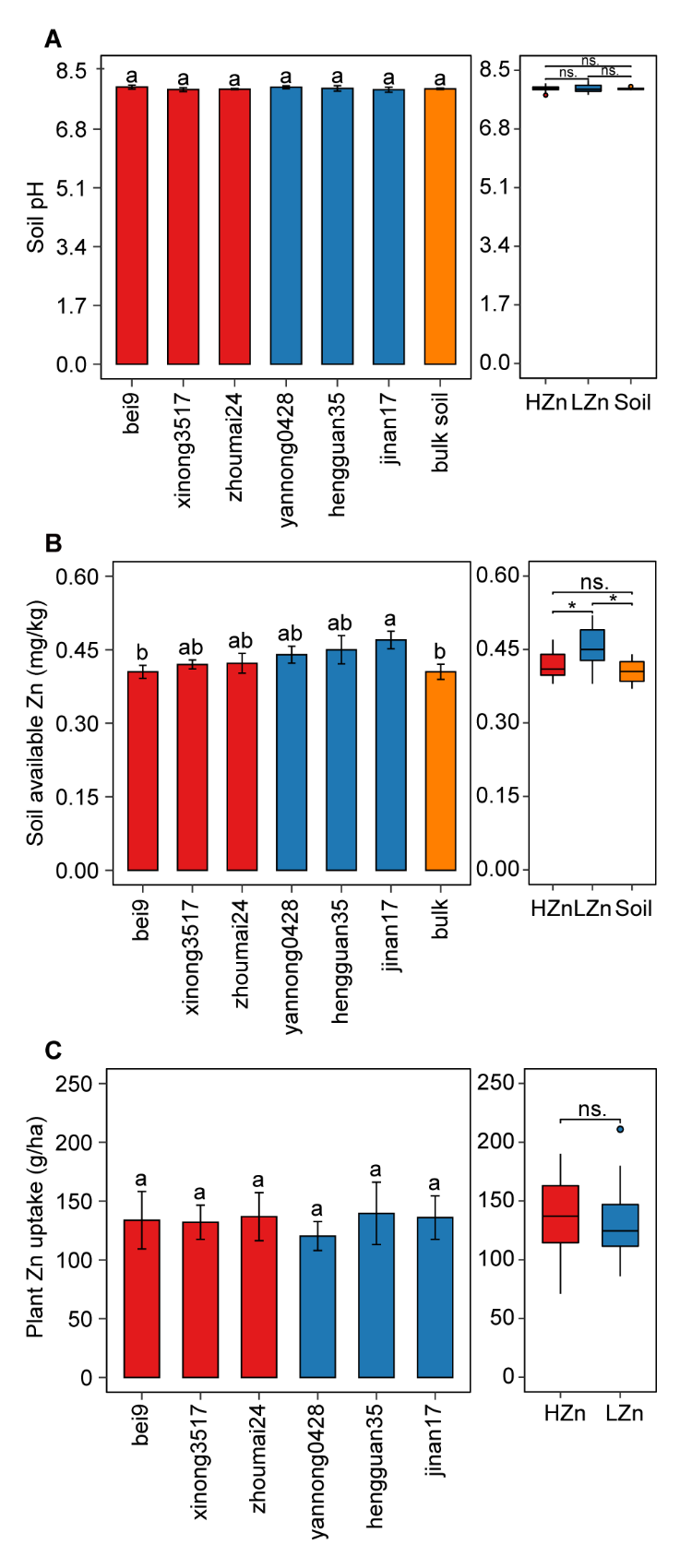


**Supplementary Figure 1.** Comparisons of rhizosphere soil pH (A), available Zn (DTPA extractable) (B) and plant Zn uptake (C) among High-Zn (HZn), Low-Zn (LZn) wheat cultivars and bulk soil (Soil). In the left panels, the error bars stand for mean ± standard error (n = 4) and different lowercase letters indicate significant differences among cultivars and Soil (ANOVA, Duncan, *P* < 0.05). In the right panels, the inner horizontal lines, bottoms and tops of boxes indicate the medians, 25th and 75th percentiles, respectively, the lower and upper whiskers extend to the minimum and maximum of the data excluding the outliers denoted by dots, and asterisks indicate significant differences among HZn (n = 12), LZn (n = 12), and Soil (n = 4) (*t*-test, ^*^ *P* < 0.05, ns. not significant).


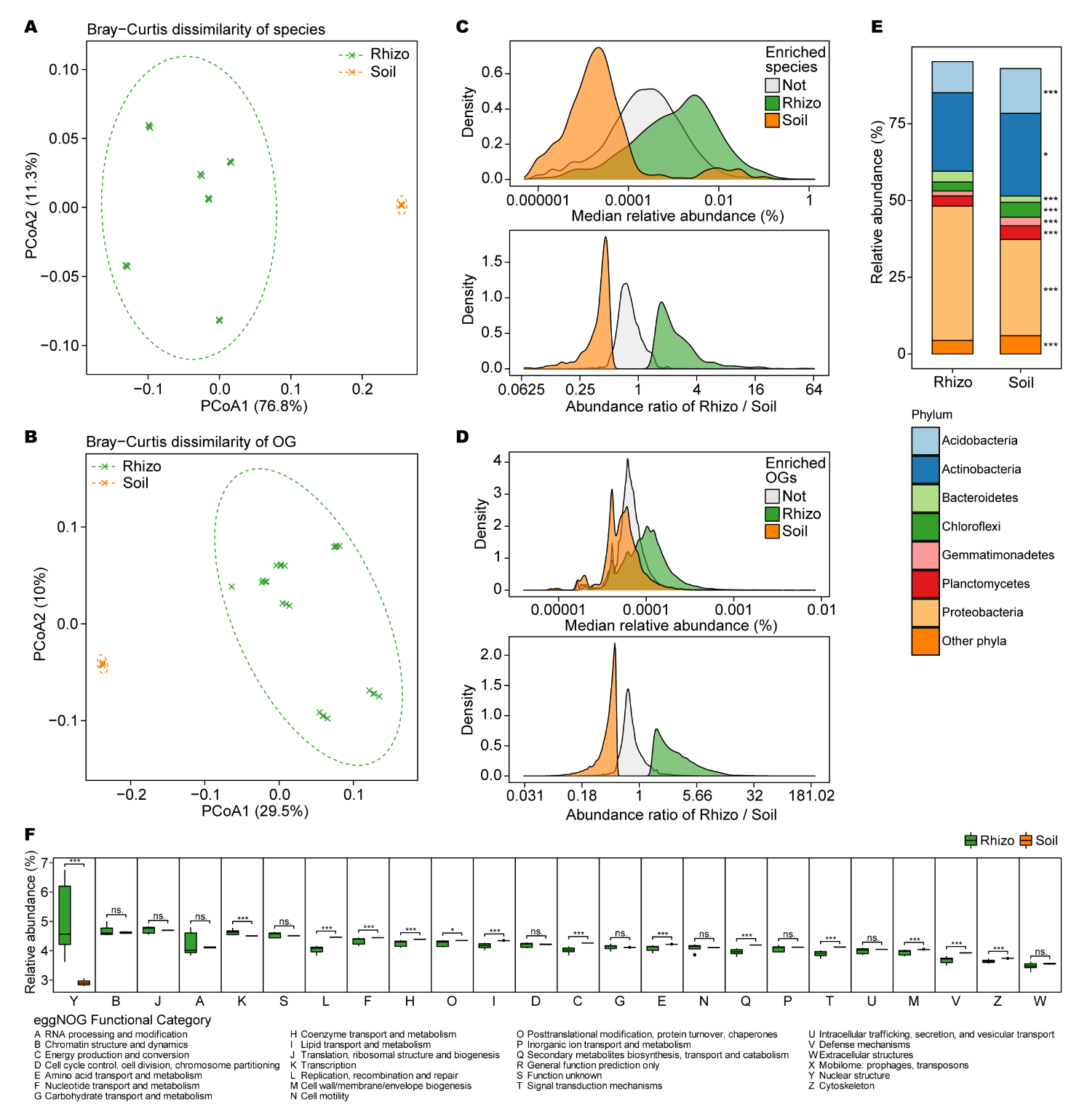


**Supplementary Figure 2.** Comparisons of microbial taxonomic and functional profiles between wheat rhizosphere (Rhizo) and bulk soil (Soil). Between-sample beta diversity of species (A) and eggNOG Orthologous Groups (OGs) (B) among Rhizo and Soil samples are analyzed by principal co-ordinate analysis (PCoA) of Bray-Curtis dissimilarity. Green and orange crosses refer to Rhizo and Soil samples and the corresponding ellipses cover 90% of the data range. Probabilistic distribution density of species (C) and OGs (D) across median relative abundances (upper panel) and abundance ratio (Rhizo / Soil, lower panel). Green, orange, and light grey areas refer to the species or OGs significantly enriched in Rhizo (ratio > 1.5), Soil (ratio < 0.5) (Wilcoxon rank sum test, BH adjusted *P* < 0.05), and neither enriched, respectively. Relative abundances of major phyla (E) and eggNOG functional categories (F) between Rhizo and Soil. In the boxplots of (F), the inner horizontal lines, bottoms and tops of boxes indicate the medians, 25th and 75th percentiles, respectively, the lower and upper whiskers extend to the minimum and maximum of the data excluding the outliers denoted by dots. Asterisks indicate significant differences between Rhizo (n = 24) and Soil (n = 4) (Wilcoxon rank sum test, BH adjusted, ^*^ *P* < 0.05, ^**^ *P* < 0.01, ^***^ *P* < 0.001, ns. not significant).


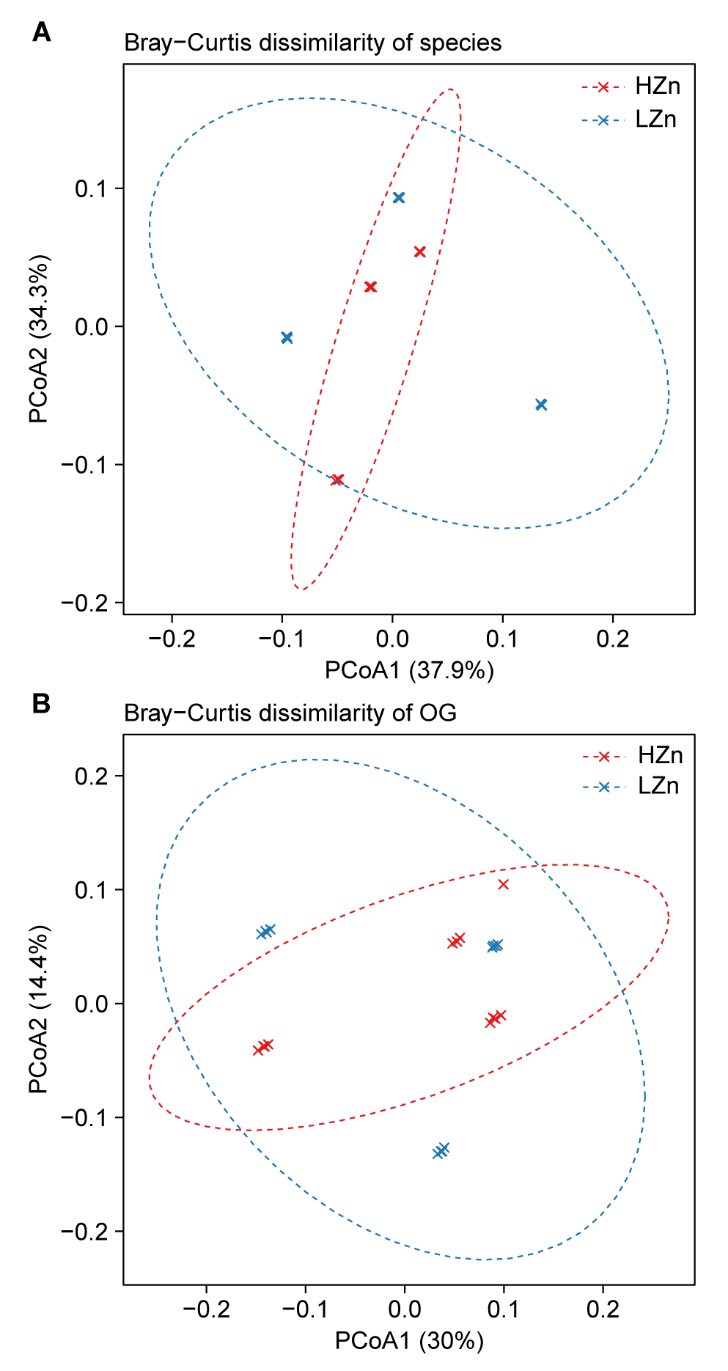


**Supplementary Figure 3.** Between-sample beta diversity of microbial species (A) and eggNOG Orthologous Groups (OGs) (B) among High-Zn (HZn) and Low-Zn (LZn) wheat rhizosphere samples are analyzed by principal co-ordinate analysis (PCoA) of Bray-Curtis dissimilarity. Red and blue crosses refer to HZn and LZn samples and the corresponding ellipses cover 90% of the data range.


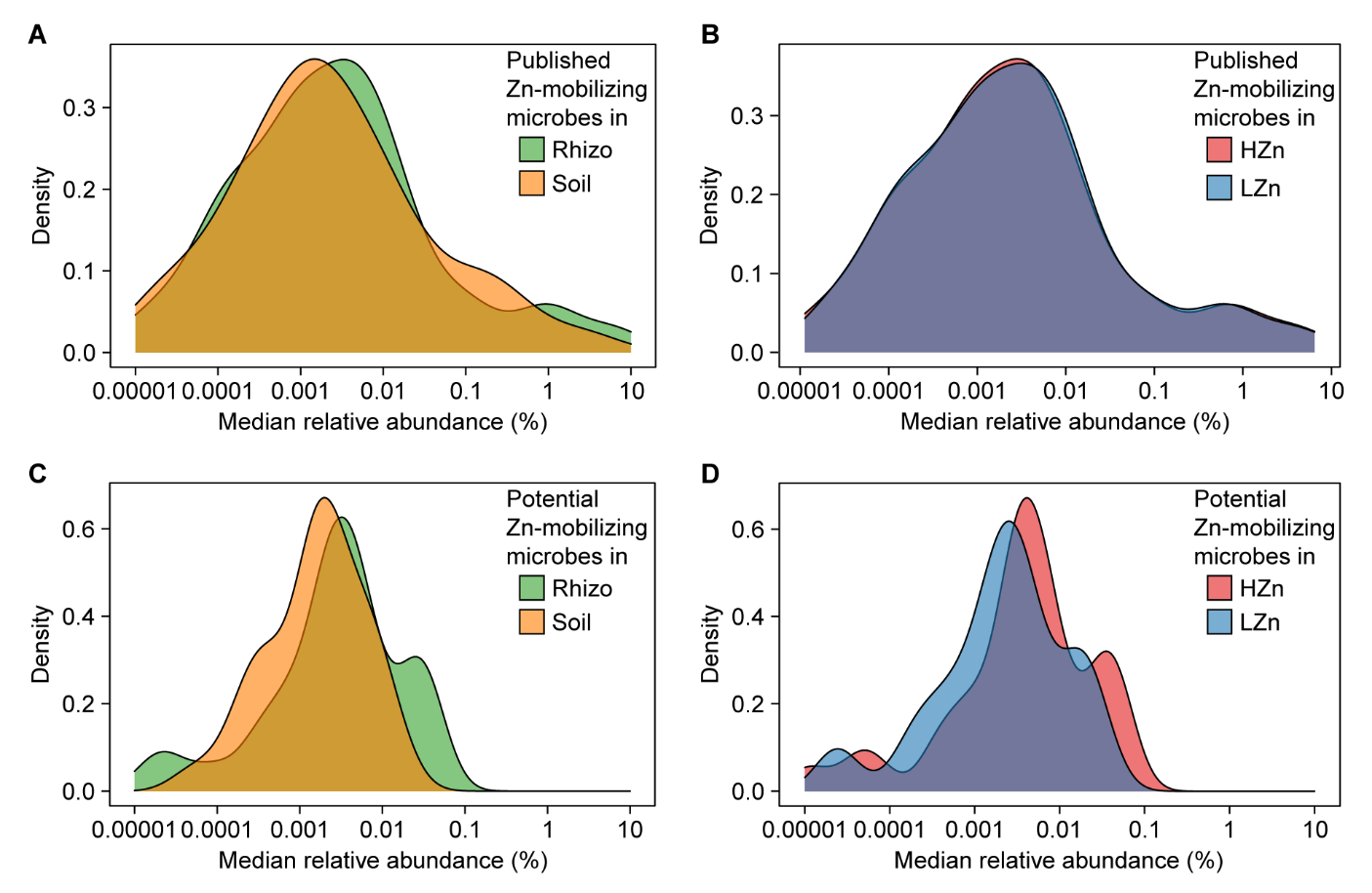


**Supplementary Figure 4.** Comparisons of the probabilistic distribution density of the median relative abundances of 38 previously published (A, B) and 30 newfound potential (C, D) soil Zn-mobilizing microbes between wheat rhizosphere (Rhizo, green) and bulk soil (Soil, orange), and between High-Zn (HZn, red) and Low-Zn (LZn, blue) rhizosphere.
